# Autonomy, disruptions and coping strategies of community-dwelling older adults in food-related activities - food shopping, cooking and eating- a scoping review

**DOI:** 10.1016/j.jnha.2025.100769

**Published:** 2026-01-08

**Authors:** Hélène Trimaille, Yoshimasa Sagawa, Aline Chassagne

**Affiliations:** a Université Marie et Louis Pasteur, INSERM, UMR 1322 LINC, F-25000 Besançon, France. UFR Santé, bâtiment Rabelais, Université Louis et Marie Pasteur, 19 rue Ambroise Paré, 25030 Besançon Cedex, France; b Centre Hospitalier Universitaire de Besançon, INSERM CIC 1431, F-25000 Besançon, France. CHU Besançon, 2 Place Saint-Jacques, 25000 Besançon, France; c Université Marie et Louis Pasteur, Department of Rehabilitation Sciences, F-25000 Besançon, France. UFR Santé, Bâtiment Rabelais, Université Louis et Marie Pasteur, 19 rue Ambroise Paré, 25030 Besançon Cedex, France; d Université Marie et Louis Pasteur, Department of Nursing Sciences, F-25000 Besançon, France. UFR Santé, Bâtiment Rabelais, Université Louis et Marie Pasteur, 19 rue Ambroise Paré, 25030 Besançon Cedex, France

**Keywords:** Food-related activities, Community-dwelling older adults, Ageing, Autonomy, Dietary knowledge, Malnutrition

## Abstract

**Background:**

Food-related activities—shopping, cooking, and eating—play a critical role in enabling community-dwelling older adults to live and age in place.

**Objectives:**

This scoping review aims to explore existing knowledge on the experiences of community-dwelling older adults in their food-related activities. It also seeks to identify which stages of these activities have been studied.

**Method:**

A systematic search of four databases (2000–2023) identified 1,189 articles. After screening, 48 studies were included. Data were analysed to determine which stages of food-related activities were examined and to extract emerging themes.

**Results:**

Of the 48 studies, 31% addressed all three stages, 14% focused on two stages, and 54% examined one stage. This review highlights the complex interplay of social, cultural, economic, political, and health environments that structure food-related activities. Disruptions within these environments challenge autonomy. Depending on their social roles, interests, skills, and knowledge, older adults develop coping strategies to maintain control over their food-related activities.

**Conclusion:**

The findings underscore the need for ethical, person-centered support from healthcare and social service professionals, as well as relatives. Support should respect the decision-making autonomy of older adults while offering tailored nutritional guidance that enables them to adapt their food-related activities to evolving needs.

## Highlights

• Most studies focus on eating stage, healthy eating.
• Food-related activities are shaped by social environment and knowledge.
• Health and social disruptions affect food-related activities and nutritional status.
• Nutritional confusion and a lack of appropriate nutritional knowledge are common.
• It is important to preserve the autonomy of older adults in all food-related activities.

## Graphical abstract

**Figure:**
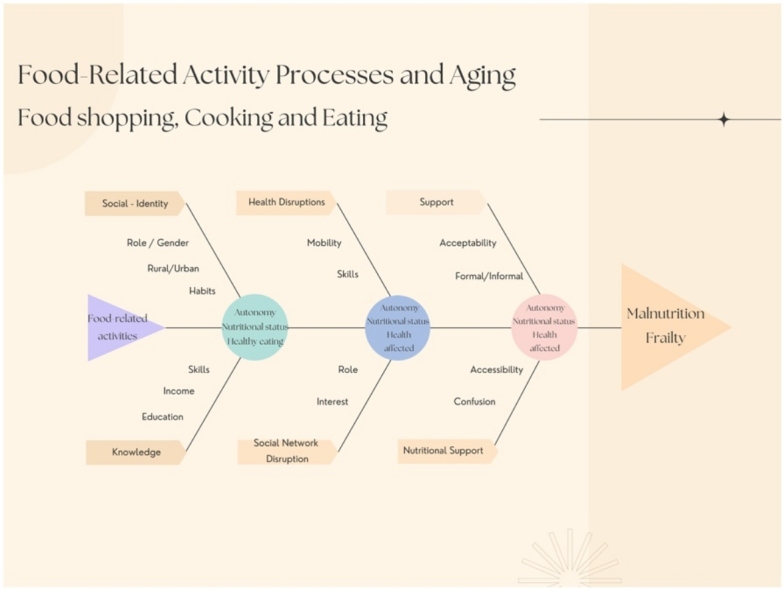

## 1 Introduction

To eat, people need to shop and prepare food, what constitute food-related activities [1]. Aging, whether normal or pathological, leads to changes at different stages of the food-related activities - namely food shopping, cooking and eating among community-dwelling older adults. Food related-activities thus play a critical role in the ability to live and age at home, a desire shared by the vast majority of older adults [2]. Studies show that protein-energy malnutrition contributes to the frailty in older adults and its associated consequences [3,4]. A meta-analysis indicates that the prevalence of malnutrition risk among older Europeans ranges from 8.5% to 28% [5]. It has been observed that older adults often lack essential nutrient intake [6] due to less varied diets and smaller portion sizes. However, research has demonstrated that adequate nutrient intake, especially through the consumption of fruits, vegetables, and proteins, helps slow brain aging, protects against pathological aging, and may reduce the onset of frailty, fall risks, and fractures. A healthy diet is seen as a critical medical intervention to prevent malnutrition, its consequences, and associated costs. Many studies focus only on improving the nutritional status of older adults with the goal of promoting "successful aging" and "healthy eating."

While it is important to "eat well" from a nutritional perspective, food-related activities cannot be understood solely through the lens of nutritional status. Indeed, malnutrition can be better understood by examining the reasons or disruptions, that prevent some older adults from consuming adequate nutrients. Aging brings about various social, health, and spatial disruptions that can affect their autonomy as well as the meaning and pleasure of maintaining food activities [7]. Some community-dwelling older adults implement coping strategies to overcome food apathy and cope with difficulties encountered at various stages of the food-related activities [8]. Through food-related activities - food shopping, cooking and eating - one perceives aspects of a person’s identity, social class, relationship with their body, values, knowledge, lifestyle and living environment [9,10]. Maintaining engagement in these food-related activities in daily life allows individuals to participate in meaningful activities, preserve their identity [1], sustain their social and gender roles [11], maintain physical and social activity, and uphold their autonomy, whether these activities are performed out of habit or genuine desire [12]. Often analysed separately or through different study designs [13], a more comprehensive understanding of the factors affecting food-related activities among community-dwelling older adults is crucial for analysing disruptions and their origins that hinder autonomy, as well as the coping strategies they adopt to maintain both their autonomy in food-related activities and their nutritional status.

The aim of this scoping review is to assess what is currently known about the experiences of community-dwelling older adults in their daily food-related activities, particularly during the three main stages of food-related activities: food shopping, cooking and eating. The second aim of this scoping review is to understand which stages of food-related activities have been analysed, and to explore the links between these stages to gain an overall understanding of the disruptions that hinder the maintenance of their autonomy in daily food-related activities and their nutritional status.

## 2 Methodology

This scoping review follows the guidelines set by the Preferred Reporting Items for Systematic Reviews and Meta-Analysis Extension for Scoping Reviews (PRISMA-ScR) [14] (See Supplemental Table A).

### 2.1 Eligibility criteria

The population of interest was community dwelling older adults. We sought studies focusing on at least one stage of the food-related activities: food shopping, cooking and eating. Studies exploring experiences, disruptions and coping strategies at different stages of food-related activities were included. Observational studies, including cross-sectional, longitudinal or qualitative designs were eligible for inclusion. Given that this study aimed to explore both representations and clinical assessments, studies analysing only nutritional status or nutritional status in one specific disease were excluded. Pilot studies, systematic reviews, Master’s or PhD theses, conferences proceedings were also excluded. Eligible studies were published in French or English from the year 2000 to February 2023 and conducted in high income countries (as defined by The World Bank).

### 2.2 Search strategy

Four databases (Web of Science, PsycINFO, PubMed and Cairn) were systematically searched for relevant literature. A predetermined list of keywords, along with relevant database-specific index terms was developed by the authors (HT, AC and YS). Due to variations in database terminology, differences in search syntax were applied across the databases. For each database, three separate searches were conducted – one for each stage of the food-related activities: food shopping, cooking and eating. Based on keywords identified in the literature, the search strategy combined various terms representing the experiences of community-dwelling older adults regarding their food-related activities. For example, we used terms such as “older adults”, “seniors”, “geriatrics” and “aging” to identify the population. We added terms like “community-dwelling”, “community-dwelling older adults”, and “independent living” to more precisely define the population of interest. Next, we applied different terms to define each stage of the food-related activities. For “food shopping” we used “meal provision”, “food access” and “food choice”. For “cooking” we used “meal preparation”, “food preparation”, “cooking skills”, “cooking practices”, “food services” and “meal on wheels”. For “eating” we used “mealtime”, “mealtime experiences” “mealtime habits”, “eating habits”, and “eating motives”. To capture their experiences, we included terms like “experiences”, “perceptions”, “attitudes”, “views”, “difficulties”, “needs”, “habits”, “behaviour”, and “routines”. The full search strategies are provided in Supplemental Table B.

### 2.3 Identifying relevant articles

Articles from each database search were exported to Zotero. These articles were then imported to Rayyan [15], where duplicates were removed. Titles and abstracts were screened by two independent reviewers (HT and AC) using the predetermined inclusion criterions. Conflicts were resolved by consensus, with additional input from reviewer (YS). Full texts of all potentially relevant articles were obtained and screened by two independent reviewers (HT and AC).

### 2.4 Critical appraisal of individual sources of evidence

An appraisal of the quality of the quantitative, qualitative and mixed-method studies was conducted using criteria derived from the Mixed Method Appraisal Tool [16]. While the quality of data was considered, no articles were excluded as risk of bias. The appraisal of the quality of articles are provided in Supplemental Table C.

### 2.5 Synthesis of results

The final data extraction form included methodological aspects as population characteristics, as well as the main findings related to food shopping, cooking, eating. To shed light on the experiences of community-dwelling older adults with their food-related activities and nutritional status, each food-related activities is examined within its broader context to highlight the influence of social environment and knowledge. This is followed by an exploration of how age-related disruptions in social and health environments affect these food-related activities, and finally how older adults cope with these changes.

## 3 Results

**Fig. 1:**
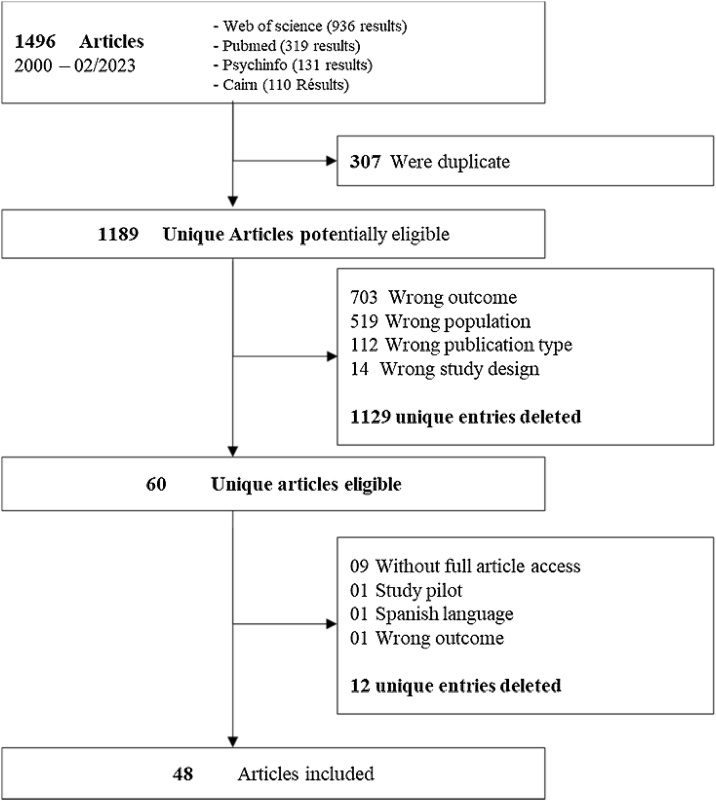
PRISMA Flow diagram.

Searches of electronic databases identified 1,189 articles, after duplicates were removed. Of them, 1129 articles were excluded due to wrong outcome (703), population (519), publication type (112) and study design (14). This left 60 eligible articles, of which 12 were excluded due to lack of access to the full text (9), wrong study design or outcome (2), and language issue (1). In the end, 48 articles were included. A PRISMA flow diagram of the study selection process is shown in Fig. 1.

### 3.1 Study characteristics

In 48 articles, 31% (15 articles) explored all three stages of food-related activities between 2003 and 2021. Among them, 13 used qualitative methodology and 2 used quantitative methodology. Ten studies were conducted in European countries, 3 in Oceania, 1 in Asia and 1 in North America. Fourteen percent (7 articles) explore two stages of food-related activities between 2001 and 2022. Among these, 4 studies were mixed-methods and 3 used qualitative methodology. Three studies were conducted in European countries, 2 in Oceania, and 2 in North America. Four studies explored food shopping and cooking, 2 studies focused on cooking and eating and 1 study analysed food shopping and eating. Finally, 54 % (26 articles) analysed only one stage of food-related activities between 2011 and 2022. Among them, 17 studies used quantitative methodology, and 9 used qualitative methodology. Sixteen studies focused on eating, 7 on food shopping, and 3 on cooking. Fourteen studies were conducted in Europe, 6 in Asia, 5 in North America, and 1 in Oceania. In total, 27 articles were from Europe, 8 from North America, 7 from Asia, and 6 from Oceania. Food-related activities are analysed through different research lenses depending on the region. In Europe, the most prominent research themes were nutritional knowledge, frailty and nutritional risk, and technology. In North America, the focus was primarily on food access and security. In Asia, the dominant theme was eating alone, while in Oceania, studies mostly explored food choice and nutritional knowledge. Topics such as nutritional risk and food access appear consistently across the entire study period, while themes like nutritional knowledge, eating alone, and technology have emerged more recently. Of the included studies, 19 studies were conducted in urban or suburban areas, 18 in both urban and rural areas, and only 3 exclusively in rural areas, 8 studies did not specify the setting. In 9 studies, participants were living independently with home care services. In 28 studies, participants are recruited regardless of their medical condition, 8 studies included only healthy participants, and 7 focused solely on community-dwelling older adults with functional disabilities, malnutrition or risk of malnutrition, 5 studies did not specify it. Study characteristics are presented in Table 1, and data research are provided in Supplemental Table D. Across studies, a wide variety of instruments was used to assess malnutrition, food frequency, diet quality, nutrition knowledge, and food-related activities, as well as cognitive status, depression, daily activity, health, frailty and social network (See Table 2).

**Table 1:** Characteristics of included studies examining one, two, or three stages of food-related activities.

N°	Authors,Year,Country	Study Design	Method	Participants (women)	Age		Residential area	Living independently/ Home help	Healthy / Medical condition	Aims	Food-related activities
					Mean age	Age					
*17*	Bloom et al., 2017, United Kingdom	Qualitative	Focus group discussions	92 (43)	78		Urban / Rural	Independent	Malnutrition	To explore influences on diet and to gain insight into gender differences and factors linked to differences in diet stability in older age.	Food shopping, Cooking and Eating
*18*	Lee et al., 2017, Taiwan	Quantitative	Questionnaire survey	564 (320)		65−76	Urban / Rural	Independent	Both	To understand Eating Competence in Taiwanese older adults.	Eating
*19*	Asamane et al., 2019, United Kingdom	Qualitative	Interviews	92 (35)	70,6		Urban / Rural	Independent	Both	To identify and compare factors influencing eating behaviours and physical function among ethnic older minorities, and to understand how these factors and their association with healthy eating and physical function changed over 8 months.	Food shopping, Cooking and Eating
*20*	Host et al., 2016, Australia	Qualitative	Focus groups discussions	18 (13)	78,2		Urban / Suburban	Independent	Healthy	To qualitatively investigate food shopping, cooking and eating habits, as well as attitudes towards the consumption of different food groups.	Food shopping, Cooking and Eating
*21*	Sidenvall et al., 2001, Sweden	Qualitative	Interviews	41 (41)		64+	Urban / Rural	Independent	Both	To study older Swedish women’s experiences of managing food shopping and cooking, as part of an independent life in different family situations.	Food Shopping and Cooking
*22*	Gomi et al., 2022, Japan	Quantitative	Online survey	1103 (576)	70		Rural	Independent	Both	To investigate the relationship between distance to the nearest food store and diet variety in rural community-dwelling elderly Japanese.	Food Shopping
*23*	Oemichen and Smith., 2016, United States of America	Qualitative	Focus group discussions	62 (49)		60+	Urban / Rural	Independent	Both	To investigate food security, food access, and food choice among free-living, lower-income seniors who lived in counties with a high and low Supplemental Nutrition Assistance Program (SNAP) participation rate.	Food Shopping
*24*	Peura-Kapanen et al., 2017, Finland	Qualitative	Empathy based stories, Focus group discussions	114 (70) 54 (31)		65+	Urban	Independent	MD	To examine how older people relate to food, and especially convenience food and convenience food packaging.	Food shopping, Cooking and Eating
*25*	Radermacher et al., 2010, Australia	Mixed	Questionnaires, Focus group discussions	37 (24)	70	58−85	Urban / Suburban	Independent	Both	To investigate the experiences and barriers to food security of community-dwelling older people.	Food Shopping and Cooking
*26*	Cardon and Gojard, 2008, France	Mixed	Questionnaires, Interviews	800−1600 (3/4) 50 (MD)		60+	Urban / Rural	Independent	Both	To investigate food-related delegation of community-dwelling older adults on their diet.	Food Shopping and Cooking
*27*	Chalermsri et al., 2020, Thailand	Qualitative	Focus group discussions, Interviews	26 (13) 13 carers (11)	71,5		Urban / Rural	Independent	Both	To explore the experiences and determinants influencing the food choices and dietary practices from both their caregivers’ and older adults’ perspectives.	Food shopping, Cooking and Eating
*28*	Chatindiara et al., 2020, New Zealand	Qualitative	Interviews, questionnaires	16 (9)	81		Urban	Both	Both	To explore older adults’ perspectives and experiences of food and nutrition intake, and to gain new insights to factors that influence vulnerability to malnutrition risk at older age.	Food shopping, Cooking and Eating
*29*	Coenen et al., 2021, Australia	Quantitative	Survey	337 (206)	82		Urban	Home help	Both	To investigate the views and expectations of current Meals on Wheels (MOW) customers.	Food shopping, Cooking and Eating
*30*	Munoz-Plaza et al., 2013, United States of America	Qualitative	Interviews, observations	30 (24)	73		Urban	Independent	Both	To identify factors involved in food shopping among older urban adults.	Food shopping
*31*	Zalega., 2020, Poland	Quantitative	Survey	2476 (1713)	74		Urban	Independent	MD	To analyse food expenditure, dietary behaviour and the frequency of food purchases in Polish silver singles’ households.	Food shopping
*32*	Payne et al., 2020, United Kingdom	Qualitative	Interviews	23 (16)		65−94	Urban / Rural	Both	Malnutrition	To explore how older adults, with health or social conditions associated with risk of malnutrition, experience psychosocial factors relevant to appetite and eating behaviour.	Food shopping, Cooking and Eating
*33*	Cardon., 2015, France	Qualitative	Interviews, observations	80 (40)		60+	MD	Independent	Both	To explore what physical and psychological disorders do to domestic gender-role assignment in food-related activities.	Food shopping, Cooking and Eating
*34*	Gustafsson et al., 2003, Sweden	Qualitative	Interviews	72 (72)	74,9		Urban / Rural	Independent	Both	To explore the cultural meaning of accomplishing food-related work by older women.	Food shopping, Cooking and Eating
*35*	Vesnaver et al., 2012, Canada	Qualitative	Observational study, Interviews	1793 (MD) 30 (24)	74,87		Urban / Suburban	Independent	Both	To identify and describe adaptive strategies, and to explore and describe the key themes of dietary resilience.	Food shopping, Cooking and Eating
*36*	Den Uijl et al., 2016, The Netherlands	Mixed	Online survey, Interviews	398 (240) 40 (20)	65,8 66.9		MD / Online	Both	Healthy	To identify consumer segments on the basis of mealtime functionality.	Cooking and Eating
*37*	Bostic et al., 2016, USA	Qualitative	Interviews	17 (14)		60+	Rural	Independent	Both	To view the trajectories of cooking throughout adulthood.	Cooking
*38*	Ellis et al., 2022, USA	Qualitative	Interviews	23 (18)	71		MD / Online	Independent	Healthy	To explore individual and interpersonal factors affecting the eating behaviours and dietary intake during the COVID-19 pandemic.	Cooking and Eating
*39*	Den Uijl et al., 2014, The Netherlands	Quantitative	Online survey	392 (234)	65,8		MD / Online	Independent	Healthy	To identify consumer segments of vital community-dwelling older persons on the basis of the emotions that they associate with their mealtimes.	Eating
*40*	Kramer et al., 2022, The Netherlands	Quantitative	Online questionnaires	32 (18)	73		MD	Independent	Both	To identify factors influencing ECAs use and if it could change dietary behaviour and decrease loneliness.	Eating
*41*	Linschooten et al., 2021, The Netherlands	Qualitative	Focus groups discussions, Interviews, Questionnaires	188 (115)	75,7		Urban / Rural	Independent	Both	To gain insights into opinion towards healthy eating and high protein products.	Eating
*42*	Krahn et al., 2011, Canada	Mixed	Focus group discussions, questionnaires	39 (25)	72,6		Rural	Independent	Both	The objective of this study was to examine the healthy eating perceptions of older adults residing in rural Manitoba, Canada.	Food Shopping and Eating
*43*	Avgerinou et al., 2019, United Kingdom	Qualitative	Interviews, Focus group discussion	24 (17) 9 carers (6).		75+	Urban / Suburban	Independent	Malnutrition	To explore the views and dietary practices of older people and their carers. To identify facilitators and barriers to healthy eating, and potential interventions.	Food shopping, Cooking and Eating
*44*	Kallio et al., 2008, Finland	Quantitative	National sample and Interviews	1697 (1071)		65+	Urban	Home help	Functional disabilities	To understand and depict the prevalence of different meal patterns.	Food shopping, Cooking and Eating
*45*	Pilleron et al., 2018, France	Quantitative	Surveys	1724 (1077)	75,7		Urban	Independent	Both	To investigate the association between dietary clusters and the 10-year risk of activity limitation in a sample of the 3C Study.	Eating
*46*	Ong et al., 2021, Singapore	Quantitative	Survey	400 (217)	71,2		Urban / Suburban	Independent	Healthy	To understand the nutrition knowledge, competencies and attitudes.	Eating.
*47*	Reynolds et al., 2021, Ireland	Qualitative	Interviews and Questionnaires	13 (6)	80		Urban	Independent	Malnutrition	To explore the lived experience of being malnourished and barriers and facilitators to management with a particular focus on ONS.	Eating
*48*	Low et al., 2020, Australia	Qualitative	Focus group discussions	16 (13) 6 carers (5)	84,2	.	Urban / Suburban	Independent	Both	To explore the experiences of community-dwelling older people living in one-person households in the ACT influence dietary patterns, food choices and perceptions about food availability.	Food Shopping and Cooking
*49*	Chang and Hickman., 2018, United States of America	Quantitative	Nationwide cross-sectional surveys	1323 (824)		65+	Urban / Rural	Independent	Both	To assess the association between functional limitations in low-income elderly adults and food insecurity and poor diet quality.	Food Shopping
*50*	Shikany et al., 2020, United States of America	Quantitative	Survey, questionnaires	5339 (5339)	78,8		Urban / Rural	Independent	Healthy	To examine whether specific social, physical, and financial factors were associated with diet quality.	Food Shopping
*51*	Fleury et al., 2021, France	Qualitative	Interviews	21 (21)	85	70−97	Urban / Suburban	Home help	Both	To explore the setting-up and the appropriation processes of meals-on-wheels service.	Food shopping, Cooking and Eating
*52*	Ishida and Ishida, 2019, Japan	Quantitative	Nationwide survey	483 (MD)		65+	Urban / Rural	Independent	Both	To clarify the relationship between dietary practices among community-dwelling older adults and family structure.	Eating
*53*	Ishida and Ishida, 2021, Japan	Quantitative	Nationwide surveys	1336 (696)		65+	Urban / Rural	Independent	Both	To examine whether eating alone has negative effects on dietary practices.	Eating
*54*	Kuoppamaki et al., 2021, Sweden	Qualitative	Video recordings of cooking task	7 (4)		65+	MD	Independent	MD	To investigate conditions and present possibilities for assistive technology to provide physical and cognitive support to older adults in cooking tasks.	Cooking
*55*	Brownie., 2013, Australia	Qualitative	Focus groups discussions	29 (23)	73		Urban / Rural	Independent	Both	To explore older people’s views about how getting older has influenced their dietary practices.	Eating
*56*	Soriano et al., 2020, France	Quantitative	Survey	1531 (980)	74		Urban / Rural	Independent	Both	To investigate if difficulties with meal-related activities can contribute to weight loss in community-dwelling older people.	Eating
*57*	Payne et al., 2021, United Kingdom	Qualitative	Interviews	41 (27)		65−94	Urban / Rural	Both	Malnutrition	To identify beliefs or contextual issues that undermined or supported older adults’ engagement with a prototype intervention to address risk of malnutrition.	Eating
*58*	Aure et al., 2020, Norway	Qualitative	Interviews	18 (12)	81		Urban	Home help	Malnutrition	To investigate older adults’ experiences of using the Appetitus app with support from healthcare professionals.	Eating
*59*	Van Wymelbeke-Delannoy et al., 2022, France	Quantitative	Surveys	878 (582)		65+	Urban / Rural	Independent	Both	To explore nutritional risk among older people with culinary dependence.	Eating
*60*	Sakurai et al., 2021, Japan	Quantitative	Questionnaires survey	710 (415)		65+	Urban / Suburban	Independent	Both	To determine whether the association between eating alone and depression symptoms.	Eating
*61*	Heinio et al., 2017, Finland, The Netherlands	Quantitative	Online survey	1221 (733)		55+	MD / Online	Independent	Healthy	To identify the salient features of ready-made meal packaging appreciated.	Food shopping.
*62*	Pajalic and Pajalic., 2014, Sweden	Quantitative	Questionnaires	274 (173)	MD	MD	Urban	Home help	MD	To explore perceptions of the food delivery municipal service.	Eating
*63*	Perotti and Strutz., 2022, Germany	Quantitative	Online survey	106 (74)	73,78		MD / Online	Independent	Healthy	To assess how older adults evaluate the AuRorA system and to collect data on actual willingness to use the technology.	Cooking
*64*	Kramer et al., 2021, The Netherlands	Qualitative	Co-design sessions, diary	13 (12)	88		Urban / Rural	Independent	MD	To describe a co-design process with older adults that informs both the content and the appearance of such an Embodied Conversational Agent.	Food shopping, Cooking and Eating

**Table 2:** Instruments used to assess food-related activities, nutritional status, physical and cognitive health and social status across included studies.

***N°***	Authors,Year,Country	Activity daily living	Health status	Cognitive status	Depression Scale	Frailty - Physical condition	Social Network / Loneliness Scale	BMI	Malnutrition Screening	Food frequency Scale	Healthy diet/ Nutrition Knowledge	Food shopping	Cooking	Eating	MOW, Ready made meals scale	Technology Usage Scale
*17*	Bloom et al., 2017, United Kingdom					Interview				FFQ	Interview	Interview				
*18*	Lee et al., 2017, Taiwan										EC Scale			Emotions, Functionnality		
*19*	Asamane et al., 2019, United Kingdom		Self-rated health			Interview		BMI						Eating behavior Interview		
*20*	Host et al., 2016, Australia							BMI	MNA							
*21*	Sidenvall et al., 2001, Sweden											Interview	Interview			
*22*	Gomi et al., 2022, Japan	Help to shop	Current medical history, Medication, Lifestyle		K6	IPAQ-SV		BMI		DVS		Food environment: Distance, In-house garden		Chewing ability	Food service use	
*23*	Oemichen and Smith., 2016, United States of America							BMI				Food security, assistance program; Interview				
*24*	Peura-Kapanen et al., 2017, Finland														Interview	
*25*	Radermacher et al., 2010, Australia	Help to shop	Health status									Food Security Survey Module; Interview	Cooking experience Interview	Factors that influence eating habits; mealtime experience Interview		
*26*	Cardon and Gojard, 2008, France											Food shopping and consumption, week diet re calls				
*27*	Chalermsri et al., 2020, Thailand	Interview	Interview	Interview								Interview				
*28*	Chatindiara et al., 2020, New Zealand	Home help	Illness severity					BMI	MNA							
*29*	Coenen et al., 2021, Australia														MOW profile	
*30*	Munoz-Plaza et al., 2013, United States of America					Interview	Interview					Interview; Assistance programs, food security	Interview	Interview		
*31*	Zalega., 2020, Poland									Consumption frequency	Sources of nutrition knowledge	Food expenditure				
*32*	Payne et al., 2020, United Kingdom	Help to shop or cook	Chronic diseases, Hospital admission, Self-rated health						Interview			Interview	Interview	Interview		
*33*	Cardon., 2015, France											Interview	Interview	Interview		
*34*	Gustafsson et al., 2003, Sweden	Interview	Interview				Interview			Dietary intake for 5 days		Interview	Interview	Interview		
*35*	Vesnaver et al., 2012, Canada		Chronic diseases	3MS		Perceived physical functionning (SF36)		BMI	Decreased appetite		C-HEI					
*36*	Den Uijl et al., 2016, The Netherlands	Assisted living	Health status								Interest, knowledge			Meal patterns, 15 EsSense Profile, Functionnality, Food fussiness		
*37*	Bostic et al., 2016, USA												Interview			
*38*	Ellis et al., 2022, USA						QAICPOA					QAICPOA				
*39*	Den Uijl et al., 2014, The Netherlands		Health attitude											Meal patterns, 15 EsSense Profile, Functionnality, Food fussiness		
*40*	Kramer et al., 2021, The Netherlands	Daily functioning	Quality of life	Mental functions		Bodily functions	Social and societal participation			Daily food diary			Daily food diary	Daily food diary		
*41*	Linschooten et al., 2021, The Netherlands							BMI		Three Day Food Record; Pro 55+	Interview			Interview		
*42*	Krahn et al., 2011, Canada							BMI	SCREEN II	Three Day Food Record						
*43*	Avgerinou et al., 2019, United Kingdom								Interview			Interview	Interview	Interview	Interview	Interview
*44*	Kallio et al., 2008, Finland	Self-rated mobility	Self-rated health											Meal patterns		
*45*	Pilleron et al., 2018, France	ADL, IADL	Medical history, Lifestyle, Multimorbidity	MMSE; DSM-IV	CES-D			BMI		24 h dietary recall; FFQ						
*46*	Ong et al., 2021, Singapore							BMI	MUST		NLQ					
*47*	Reynolds et al., 2021, Ireland	EuroQOL EQ-5D-5L	EuroQOL EQ-5D-5		EuroQOL EQ-5D-5				Interviews							
*48*	Low et al., 2020, Australia			MMSE			Interview		MNA			Food choice, security interview		Interview		
*49*	Chang and Hickman., 2018, United States of America	Functional limitations	Chronic health care							Dietary assessment	Healthy diet	Food insecurity				
*50*	Shikany et al., 2020, United States of America	Help to shop or cook								FFQ	HEI-2010	Food security		Meal patterns; Eating style; Tooth or mouth problems		
*51*	Fleury et al., 2021, France	Interview		MMSE										Interview	MOW profile; Interview	
*52*	Ishida and Ishida, 2019, Japan		Self-rated health								Healthy diet			Emotions		
*53*	Ishida and Ishida, 2021, Japan										Healthy diet			Eating style (alone, with someone)		
*54*	Kuoppamaki et al., 2021, Sweden												Interview			
*55*	Brownie., 2013, Australia		Medical history, Lifestyle									Food choice Interview		Eating behavior Interview		
*56*	Soriano et al., 2020, France	ADCS ADLPI Scale.		MMSE	GDS	Frailty status; SPPB		BMI	Weight loss							
*57*	Payne et al., 2021, United Kingdom	Help to shop or cook	Health condition self report, Self-rated health, Hospital admission					BMI	MUST; SNAQ		Interview					
*58*	Aure et al., 2020, Norway	Help to shop or cook						BMI	MNA							Interview
*59*	Van Wymelbeke-Delannoy et al., 2022, France		Comorbidities	MMSE	GDS				MNA							
*60*	Sakurai et al., 2021, Japan		Subjective health, Comorbidities, Medications, Lifestyle	MMSE	SDS		LSNS-6	BMI						Eating style (alone, with someone)		
*61*	Heinio et al., 2017, Finland, The Netherlands		Health status									Ready-made meal food shopping				
*62*	Pajalic and Pajalic., 2014, Sweden														Food delivery Service	
*63*	Perotti and Strutz., 2022, Germany	Degree of disability														Technology Usage Inventory; Technology usage
*64*	Kramer et al., 2022, The Netherlands		Quality of life, Health conditions, eHealth Literacy Scale		BPNSFS		De Jong Gierveld Loneliness scale		MNA	Previous day’s fruit, vegetable, liquid intake						Technology usage

Legend: **ADL:** Activities of Daily Living; **IADL:** The Lawton Instrumental Activities of Daily Living Scale; **EuroQOL EQ-5D-**5L: European Quality of Life 5 Dimensions 5 levels; **ADCS ADLPI**: Alzheimer's Disease Cooperative Study-Activities of Daily Living Prevention Instrument; **MMSE**: Mini Mental State Examination; **3MS**: Modified Mini Mental State Examination; **DSM-IV**: Diagnostic and statistical manual of mental disorders; **SDS:** Self-Rating Depression Scale; **K6**: Kessler Screening Scale for Psychological Distress; **CES-D**: Center for Epidemiological Studies-Depression scale; **GDS**: Geriatric Depression Scale; **BPNSFS:** Basic psychological need satisfaction and frustration scales; **SF36**: Medical Outcomes Study Short Form 36; **IPAQ-SV**: Physical activity level; **SPPB**: Short Physical Performance Battery; **QAICPOA**: Questionnaire for Assessing the Impact of the COVID-19 Pandemic on Older Adults; **LSNS-6**: Lubben Social Network Scale; **BMI**: Body Mass Index; **SCREEN II**: Seniors in the Community: Risk Evaluation for Eating and Nutrition; **MNA**: Mini Nutritional Assessment; **MUST**: Malnutrition Universal Screening Tool; **SNAQ**: Short Nutritional Assessment Questionnaire; **FFQ**: Food Frequency Questionnaire; **Pro 55+**: Protein screener 55+; **DVS**: Dietary Variety Score; **EC scale**: Eating Competence scale; **HEI-2010**: Healthy Eating Index; **C-HEI**: Canadian Healthy Eating Index; **NLQ**: Nutrition Literacy Questionnaire.

### 3.2 Environment and knowledge background of food-related activities

#### 3.2.1 Social environment and food-related activities

Older adults emphasized the importance of being able to choose familiar products, shaped by upbringing and past experiences [17,18]. Many combined supermarkets with smaller outlets to access familiar and traditional foods which help them feel comfortable [[19], [20], [21]], while rural community-dwelling older adults faced limited variety due to distance from stores [22] but often relied on gardening as an economical and empowering source of fresh food [[22], [23], [24], [25]]. Financial constraints strongly influenced purchasing decisions, food security [21,23,[25], [26], [27], [28], [29], [30], [31]]. Older adults reported using special store offers stores [24], purchasing in bulk, stockpiling good for future use [20,21,23,28,32], food coupons, or food pantries to access food 23,30]. Gender differences emerged, for women, food shopping could serve as an opportunity to spend time for themselves [26], while for men, it often reinforced their role in the grocery shopping task [33,34]. Cooking was particularly valued by women, considered as a priority [17,34], they expressed a strong desire to continue cooking [21,35] because it is strongly tied to “identity” and “heritage”, providing both a connection to the past and a sense of comfort [17,19,20,24,27,33]. Cooking create order in their everyday lives [33,34], preserve their nurturing role, particularly by maintaining eating routines for their husbands [20,26,33], and also perceived as economical [24]. Many women considered cooking as an enjoyable pastime [20,24,[34], [35], [36]] utilizing their skills and experience [20,37], and this autonomy boosted their self-esteem [21]. Men tended to view cooking more negatively, often seeing it as a chore and a waste of time [32,33]. However, some older adults described how improving their cooking skills and trying new recipes became a form of entertainment or a hobby [[35], [36], [37], [38]]. Some value the shift toward more casual and regular eating patterns after retirement, no longer constrained by work routines [19,32], and eat primarily for enjoyment [17,35,36,39]. They also report eating at fixed, regular times [33,34,40,41], with meal patterns - breakfast, lunch, dinner – generally maintained. These patterns reflect long-standing habits [20,28,32,33,[42], [43], [44]], and are independently associated with higher education levels and income [43,44].

#### 3.2.2 Knowledge and food-related activities

The social environment of community-dwelling older adults influences their behaviours in food-related activities. Nutrition knowledge and the perception of health aspect of food, are also shaped by this environment and, in turn, determines variations in food-related activities. Although inadequate dietary habits can significantly impact the frailty process [45], some older adults had never prioritized the health aspects of food [18,24,35,36]. Perceptions of healthy eating varied according to sex, ethnicity, medical conditions, religion, age and educational background [19,46]. Levels of “eating competence” were also influenced by lifestyle and place of living [18]. Older adults often turn to informal sources for nutritional information, such as online resources, magazines, or advice from children [31,47]. Fear of losing autonomy motivates some to maintain a nutritious diet and physical function [19,36,39,46]. Healthy eating was commonly defined not only in terms of nutrients but also by eating ‘balanced’, ‘regularly’, ‘variedly’ [20,42], ‘in moderation’ [40,42], and satiation [19]. Finally, rituals such as properly setting the table [36,41], were described as strategies to promote “conscious” and “mindful” eating [40]. Older adults reported their food choices had become much healthier [20], with an increasing focus on a balanced diet to maintain their health [35,36,39,40]. The quality and taste of food was fundamental, with older adults purchasing fresh produce [17,26,27,33,34], local produce [42,48] even when the cost was high [20,28]. Rural participants expressed a preference for ingredients they had either gathered from nature or grown themselves [24,34]. Food shopping by incorporating walking to the shops into their daily routine promoted physical activity [[19], [20], [21],^24^,^30^]. Cooking also serves as a way for women to maintain their physical and mental well-being 24]. Women considered home-cooked meals to be healthier [19,28], they often used healthy cooking methods to reduce salt or fat intake [19,20,28]. To preserve their independence, some older adults were willing to work on improving their physical capabilities [24], even if it meant spending more time in the kitchen [23,34,37].

### 3.3 Health and social disruptions in food-related activities

Thus, social background influences nutritional knowledge and shapes behaviours related to food-related activities. However, with advancing aging, these activities are increasingly affected by physical and/or cognitive limitations, as well as disruptions in social environment. Older adults aged 80 and above experience food-related difficulties more frequently than those aged 65–79 years [44].

#### 3.3.1 Food shopping: Reduce access to food and others

The level of physical functioning and degree of independence significantly influenced both their ability to shop and their food choices [17,25,26,28,30,32,35,40,43,49], which in turn impacted their diet quality [50]. Physical limitations – such as difficulty carrying bag [20,21,30,44,51], walking impairments [33], balance problems or difficulty handling a walker [48,51], pain [19], vision problems [33,42[48] - restricted autonomy in their food shopping habits [21,34], and reduced opportunity for social interaction [21]. Alongside physical limitations, changes in social environment impact food shopping. Living with a spouse, or relatives, appears protective against food insecurity and poor diet [49,52,53]. Indeed, older adults who did not have access to their own transportation had less flexibility in their grocery shopping practices [21,35]. Many older adults are forced to reduce the scale of their shopping trips to avoid physical strain, opting to shop more often but in smaller quantities [20,21,23,34]. Moreover, with loss of a spouse, especially when the spouse had been the primary driver, left some older adults dependent on local shops [21,25], if available [17]. Finally, some older adults struggled to purchase adequate amounts of food, especially when living alone, due to widowhood [17,20,42,48].

#### 3.3.2 Cooking: Reduce roles and tiredness

Cooking tasks are impacted by the shifts in personal priorities and reduced physical and/or cognitive capability to carry out cooking tasks [17,23,37,43]. The level of functioning and degree of independence influenced cooking [23,37,40,43]. Older adults reported that pain, walking difficulties, and mobility challenges hindered their ability to cook, especially when they could no longer stand or move around the kitchen comfortably [19,25,28,32,33]. Cognitive impairments also posed barriers to cook [43]. While, older adults have had more time for cooking since retirement [17,19,37], commonly they expressed that cooking had become costly and time-consuming [27,33,37] because of difficulties with basic tasks such as chopping, cutting, peeling and lifting [21,33,34,54]. Alongside these physical and/or cognitive limitations, social changes, particularly widowhood, alter the meaning of cooking. For many women, the loss of the nurturing role diminishes motivation to cook for themselves [17,19,40,55]. This often results in a reduction in both the quantity and variety of meals prepared [17,19,20,25,26,28,32,37,43]. Men, especially those whose wives previously managed cooking, report greater difficulties and frustration [33,35,44,46,51]. Poor cooking skills further undermine resilience and autonomy [33,35,43,51]. Illness or spousal loss frequently marks a turning point, influencing their ability or motivation to start—or continue—cooking [17].

#### 3.3.3 Eating: Appetite decline and social disruption

Unhealthy dietary patterns may contribute to increase risk of functional decline and activity limitation in later life [45]. While many older adults recognize the importance of healthy eating to preserve independence [20,35], knowledge gaps persist. While most older adults identified fruits and vegetables as key components of a healthy diet, they were often unaware of the importance of dietary protein [36,41], and often lack the appropriate nutritional knowledge and guidance on what constitutes a nutritious diet in later life [28,35,42,43,52,53]. Disparities between perceived and actual dietary practices reflect limited nutritional guidance and inconsistent health messaging [42]. Clinician advice is generally accepted when clearly justified [36,40,43], but dietetic referrals remain scarce, contributing to under recognition of malnutrition [47]. Moreover, older adults reported general satisfaction with their appetite, but many observed a decrease in portion sizes [17,20,43,55,56] and noted a progressive decline in appetite, reduced desire for food [17,28,32]. They often failed to view reduced intake as problematic, framing it instead as an normal and reasonable adaptation [28,32,43] to decreased physical activity [17,19,28,32,43]. Lifelong exposure to shifting and often conflicting food messages left many older adults uncertain about what constitutes appropriate food choices in later life [55,57]. Eating between meals were rare [28], and often considered inappropriate or unnecessary [19,43], as guilty [39,57]. In some cases, appetite or weight loss was even perceived positively, reflecting cultural or personal valuations of thinness and health benefit 19,32,42,57]. This resistance to nutritional guidance was particularly evident when it came to dietary recommendations for frequent eating and high-energy foods, as many were reluctant to gain weight [58]. Appetite loss was also attributed to digestive discomfort, medication, poor dentition, chewing difficulties, and diminished taste or smell [17,19,20,28,32,41,51,55,57]. Prescribed diets, whether personal or spousal, further restricted food choices by reducing portions, adjusting meal timing or avoiding certain foods [19,32,55,57], while oral nutritional supplements (ONS) were often avoided due to negative associations with illness or terminal decline, and due to difficulties with digestion [32]. Social changes play also a decisive role, loneliness and widowhood were also frequently linked to a loss of appetite, meal skipping [27,40,52] and reduce conventional meal patterns [32,44], or routines such as setting the table [34]. Eating alone, particularly among men, was correlated with reduced diet variety [50,53] and depressive symptoms [59]. Furthermore, older adults with functional limitations who lived alone were three times more likely to perceive their diet as of poor quality compared to those without such limitations [49]. Participants described various approaches to counteract eating without pleasure or desire, such as eating at set times, creating a conducive atmosphere [32,35,38]. Older adults described how companionship made meals more pleasurable [19,20,28,36,52]. Commensality or having a supportive community environment enhanced enjoyment, diet diversity, and mental health [17,19,28,32,40,52,53,60]. Residential facilities provide more opportunities for shared social contact during mealtimes, which may be lacking for those living independently [20]. However, eating alone is not universally harmful for all older adults, its negative effects on diet depend also on social context, particularly social isolation which negatively affects mental health [60]. For some women who modified their eating techniques to maintain independence [34], social eating became uncomfortable due to eating difficulties and feelings of shame, and social withdrawal emerged as a common adaptation strategy [32,34].

### 3.4 Support and its impact on food-related activities

With aging, the emergence of health conditions and the disruption of social networks generate tensions between the desire to maintain autonomy and the necessity of accepting support in food-related activities. This negotiation process directly influences nutritional status. Greater reliance on assistance for food-related activities among older adults is associated with an increased risk of malnutrition or being malnourished, and the impact varies depending on the status and role of the person to whom tasks are delegated [59].

#### 3.4.1 Food Shopping: Negotiating independence

Adjustments around accessibility and delegation were also employed to retain independence and varying with social network size. First, they made deliberate choices to purchase easy-to-open packaging, clear labelling [24,61], and foods requiring minimal preparation [26,29,33,40,48,51], and though convenience food was often seen as inferior in experience [24], it was also perceived as empowering when selected independently [34,42]. Assistive tools were valued, particularly by women, though some reported feelings of shame that limited their use [34]. Then, when older adults were no longer able to shop independently, support from family members—spouses [33,34], children [26,33], or friends—was essential in ensuring continued access to culturally or personally significant food items [19,21,26,[32], [33], [34], [35]], but often limited independent food shopping [35]. For those without relatives, transportation services and grocery delivery were highly appreciated [22,23,30,34], enabling continued access to shops and preserving autonomy in food shopping and meal preparation [29].

#### 3.4.2 Cooking: Balancing control, skills and delegation

The willingness to accept help for cooking tasks often depends on the individual skills and interests of community-dwelling older adults. Many women found it difficult – even frightening – to imagine losing the ability to cook independently [24,25,32,34,35]. Dependence on children or social services was often resisted [34,48,51], as home-cooked meals were perceived as tastier and healthier [19,32]. They strongly emphasized having always prepared their own meals and the use of partially or fully prepared meals was sometimes viewed as guilty 32] or violating deeply held personal values around food and autonomy in decision making [24,32,34,51,62]. Some men learned to cook [37], frequently under the guidance of their wives [33,34]. Strategies to maintain culinary “habitus” [34], included simplifying recipes [17,21,26,29,[32], [33], [34],^37^,^51^], adapting kitchen environments, and using tools or appliances [26,33,34,37] to reduce physical burden. Techniques such as spreading cooking tasks throughout the day [34,37,40], or reducing the frequency of cooking by preparing one-pot meals and dividing them into smaller portions for freezing [17,20], or using frozen and vacuum-packed foods [[26], [27], [28],^33^,^40^,^43^], were adopted to minimize effort while ensuring variety and reducing waste [17,20]. Delegation of cooking tasks generated tensions, particularly when older adults felt replaced rather than supported [26,33,35,51]. Assistance from relatives [19,30,32,33] was preferred over institutional services, as it preserved household roles and self-determination 34]. Meal delivery services were often introduced after hospitalizations or recognition of declining capacity [29,51]. Their adoption required reconfiguration of routines and was influenced by financial stability, access to information, and available formal support [35,51]. Autonomy was preserved when older adults could adjust the number and type of meals received, combining delivered meals with self-prepared food [24,29]. Assistive technologies could provide both physical and cognitive support in meal preparation at home [54]. While interactive voice-controlled robots were rated moderately acceptable, intention to use remained low [63]. Financial constraints limited access to advanced technologies among lower-income groups, and skepticism toward robotics or conversational agents (ECAs) was common [40,63,64]. Dietary apps, when supported by healthcare professionals, were more positively received. Visualization of nutrient and fluid intake increased dietary awareness and variety [58].

#### 3.4.3 Eating: Resilience through supportive network

Informal support networks played a crucial role in emotionally supporting older adults, helping them remain resilient eaters, and ensuring both nutritious and varied diet [18,27,32,35,47]. Meal services were often framed not as dependency but as strategies to maintain independence and control [23,35], and diet quality [20]. Congregate meal programs were valued for their affordability [23], and public food assistance programs improved diet quality among low-income older adults [50]. Older adults implemented meals delivery services they often express satisfaction [62], despite irregular delivery schedules disrupting established mealtime routines or causing missed meals [34], and a lack of assistance with tasks like reheating food [29]. Moreover, meal delivery services [23,29,51,62] or congregate meals [23] can provide social interactions to older adults. However, older adults reported that congregate meal programs were not easily accessible [42], and some men who lived or ate alone were often the most reluctant to engage in community meals initiatives [53].

## 4 Discussion

This scoping review aims to synthesize current knowledge on the evolution of food-related activities among community-dwelling older adults across the three main stages of food-related activities: food shopping, cooking and eating. It also examines how these stages are studied in existing literature. One-third of the selected studies investigate all three stages, primarily using qualitative methodologies that allow for a holistic understanding of daily food-related activities. About 10% of studies address two stages, most frequently examines food shopping and cooking, using qualitative or mixed-methods approaches. Over half of the studies focus on a single stage, predominantly analysing disruptions in eating, then in food shopping, and to a lesser extent, cooking, using mostly quantitative methods.

Healthy eating is widely associated with successful aging and the ability to age in place [19,20,35,36,39,42,[44], [45], [46],^64^]. The findings underscore how access to food, to nutritional knowledge and skills is unequal because it depends on the social, economic and cultural environment in which community-dwelling older adults have lived and continue to live. Moreover, with aging these established food-related activities are vulnerable to disruptions in social and health environments, which may be cumulative, affecting autonomy and nutritional status of community-dwelling older adults. Accordingly, a systematic yet holistic perspective on food-related activities – food shopping, cooking and eating in relation to their ecologic contexts — is crucial to understand how these food-related activities evolve and how they ultimately influence the nutritional status and the ability to age in place for community-dwelling older adults. Food-related activities are intrinsically linked to the need to the processes of shopping, cooking, and eating familiar products [[17], [18], [19], [20],^24^,^27^,^33^]. These food related activities are shaped by the social, cultural, and economic contexts, which in turn determine individual’s roles, interests, knowledge, and skills. Consequently, throughout life, the relationship between diet and health is not consistently recognised, nor can it always be prioritised by everyone [18,24,35,36]. Thus, as individuals age and in response to concerns about losing their autonomy, some older people who are aware of the relationship between diet and health may adopt practices aimed at maintaining a healthy diet in order to support healthy aging [20,35,36,39,40]. However, with advancing aging, community-dwelling older adults often experience physical and/or cognitive decline, as well as changes in their social environment. These factors may overlap and jointly affect their daily food-related activities. In the context of food shopping, health and social challenges can limit mobility and reduce access to familiar products, and compromise independence in decision making [21,34,35,50]. With regard to cooking tasks, health and social difficulties may affect individual’s role and the meaning attributed to cooking, and their ability to cook [17,19,23,33,35,37,40,43,55]. Finally, during mealtimes, the health and social challenges they face can negatively affect their appetite [17,19,20,27,28,32,40,41,51,52,55,57]. Current research [65] emphasizes the need to examine not only the adverse effects of eating alone but also the size of social networks and the social roles of community-dwelling older adults [60,66,67], in order to understand their influence on dietary resilience [19,17,51,44,18,47,35,32,60]. Compounding this, a lack of regular nutritional monitoring and inconsistent dietary recommendations—often conflicting with prior nutritional guidance—further contribute to nutritional confusion [18,19,28,35,36,[40], [41], [42], [43],[45], [46], [47],^52^,^53^,^57^]. Perceiving a decline in appetite as a normal and positive aspect of aging may hinder their ability to maintain an appropriate diet [19,32,42,57]. The ability and willingness to delegate depends on individual background and disruptions in health and social environments, which determine individual abilities, skills, interest, knowledge and access to support for food-related activities. When community-dwelling older adults acknowledge the need for help, for some following hospitalization, the impact of support on their nutritional status appears to be greater when they are entirely replaced in their food-related activities rather than accompanied, as these activities then no longer reflect their own habits, preferences [26,33,35,51,59]. The use of digital tools to enhance nutritional knowledge and autonomy [54,58] remains limited and depends on both professional support and the older adult’s familiarity, financial capacities and comfort with technology [63,64]. Community-dwelling older adults, according to their knowledge, interest and skills, adapt their food-related activities in response to these changes, often by reducing their engagement while maintaining a certain “habitus” [34] to preserve their identity and independence, retain control over their food-related activities, and support healthy aging [29,34,42]. Promoting autonomy in food-related activities and empowerment through tailored support that integrates nutritional guidance [[68], [69], [70]] represents a key lever for sustaining food-related activities and supporting aging in place. A deeper understanding of how older adults manage, adapt, and delegate food activities is crucial for designing ethically sound interventions that respect individual needs and preferences.

Although the scoping review focused on the food-related activities of community-dwelling older adults, included articles examined heterogeneous populations varying in age, gender, health status and levels of autonomy. The database search strategies were based on a wide range of keywords identified in the literature. However, these likely do not capture the full spectrum of relevant articles, reflecting the lack of consensus around definitions related to both the various stages of food-related activities and their experiences. The included articles employed diverse data collection methods and a wide range of standardized and non-standardized scales, often without systematic application. It would therefore be appropriate to consider a unified tool that addresses the multiple dimensions of malnutrition risk, enabling both comprehensive assessment of the phenomenon and the effective prevention.

## 5 Conclusion

This scoping review aimed to analyse food-related activities of community-dwelling older adults. The finding reveal that most studies focus on eating stage, often emphasizing the concept of 'healthy eating'. While healthy eating is indeed a key component of successful aging, it is shaped by social environment of community-dwelling adults. Consequently, depending on individual resources, healthy eating is not always a priority, and it becomes even more challenging when health and social changes associated with aging intersect. Analysing it in isolation overlooks systemic disruptions that may occur at different stages and accumulate across multiple environments. By examining disruptions and coping strategies developed by community-dwelling older adults in their food-related activities, this scoping review underscores the critical importance of preserving autonomy across all stage of food-related activities. Maintaining this autonomy is essential not only for nutritional well-being but also for sustaining identity, social roles, sense, as well as for supporting aging in place. Given the interdependent nature of food-related activities, future research should adopt qualitative, process-oriented approaches to better understand disruptions, coping strategies, and effective way to support community-dwelling older adults without undermining their autonomy in decision-making. It is therefore essential to question not only the impact of eating alone but also the broader effects of a shrinking social network and the evolving social status of community-dwelling older adults. At the same time, the literature reveals significant confusion regarding nutritional guidelines for community-dwelling older adults. Thus, these findings are relevant to a wide range of stakeholders - including policymakers, social workers, health professionals (nurses, general practitioners, dietitians, nutritionists), community-dwelling older adults and their relatives. These findings highlight the need for ethically grounded support that involves both health and social care professionals as well as relatives. Such support must uphold the autonomy of older adults, providing adequate nutritional guidance and support that enables them to maintain familiar habits, adapted to their changing needs.

## CRediT authorship contribution statement

**HT**: Conceptualization, methodology, formal analysis, investigation, writing – original draft/reviewing and editing, supervision. **AC:** Methodology; formal analysis, writing – original draft/reviewing and editing, supervision. **YS:** Methodology, formal analysis, writing- original draft/reviewing and editing, supervision. All authors have seen and approved the final version of the manuscript.

## Ethical statement

As this study is a review, approval from institutional ethics committee was not required.

## Funding

This work is part of a thesis carried out as part of the CARAVANE research project that was supported by the French National Research Agency[ANR-22-CE55-0001, 2022].

## Declaration of competing interest

The authors have no conflicts of interest to declare.
