# Supplementary material for: Autonomy, disruptions and coping strategies of community-dwelling older adults in food-related activities - food shopping, cooking and eating- a scoping review
Source: J Nutr Health Aging. 2026 Jan 8;30(2):100769. doi: 10.1016/j.jnha.2025.100769 (PMC12816844; doi:10.1016/j.jnha.2025.100769)
Supplement: Supplementary file 2 [file mmc2.docx]

Search strategies

1. Web of Science strategy

The Web of Science search strategy was as follows: TX (older adults or elderly or seniors or geriatrics or aging) AND TX (Community-dwelling or Community-dwelling older adults or Independent Living) OR TX (Cooking or Meal preparation or Food preparation or Cooking skills or Cooking practices or Food Services or Meal on Wheels) OR TX (Mealtime or Mealtime experiences or Mealtime habits or Eating habits or Eating or Eating motives) AND TX (Food shopping or Meal provision or Food access or Food choice) AND TX (experiences or perceptions or attitudes or views or difficulties or needs or habits or behavior or routines)

1. Psychinfo search strategies

Psychinfo search strategy for food shopping was a follow: TX (older adults or elderly or seniors or geriatrics or aging) AND TX (Community-dwelling or Community-dwelling older adults or Independent Living) AND TX (Food shopping or Meal provision or Food access or Food choice) AND TX (experiences or perceptions or attitudes or views or difficulties or needs or habits or behavior or routines).

For cooking: TX (older adults or elderly or seniors or geriatrics or aging) AND TX (Community-dwelling or Community-dwelling older adults or Independent Living) AND TX (Cooking or Meal preparation or Food preparation or Cooking skills or Cooking practices or Food Services or Meal on Wheels) AND TX (experiences or perceptions or attitudes or views or difficulties or needs or habits or behavior or routines).

And finally for Eating: TX (older adults or elderly or seniors or geriatrics or aging) AND TX (Community-dwelling or Community-dwelling older adults or Independent Living) AND TX (Mealtime or Mealtime experiences or Mealtime habits or Eating habits or Eating or Eating motives) AND TX (experiences or perceptions or attitudes or views or difficulties or needs or habits or behavior or routines). The three requests were then combined using the Boolean operator “OR”.

1. Pubmed search strategies

Pubmed search strategy for Food shopping (("older adults"[TW] OR "elderly"[TW] OR "seniors"[TW] OR "geriatrics"[TW] OR "aging"[TW] OR Aging*[MH] ) AND ("Community-dwelling "[TW] OR "Community-dwelling older adults "[TW] OR "Independent Living*"[TW] OR Independent Living*[MH] ) AND ("Food shopping "[TW] OR "Meal provision "[TW] OR "Food access "[TW] OR "Food choice"[TW] OR Food preferences*[MH] ) AND ("experiences"[TW] OR "perceptions"[TW] OR "attitudes"[TW] OR "views"[TW] OR "difficulties"[TW] OR "needs"[TW] OR "habits"[TW] OR "behavior"[TW] OR "routines"[TW] OR Perception*[MH] OR Habits[MH] ) AND (2000/01/01:3000/12/31[dp])) NOT ("animals"[MH] NOT "humans"[MH]) NOT (Mice[MeSH] OR "Models, Animal"[MH])

Pubmed search strategy for Cooking (("older adults"[TW] OR "elderly"[TW] OR "seniors"[TW] OR "geriatrics"[TW] OR "aging"[TW] OR Aging*[MH] ) AND ("Community-dwelling "[TW] OR "Community-dwelling older adults "[TW] OR "Independent Living*"[TW] OR Independent Living*[MH] ) AND ("Cooking "[TW] OR "Meal preparation "[TW] OR "Food preparation "[TW] OR "Cooking skills "[TW] OR "Cooking practices "[TW] OR "Food Services"[TW] OR "Meal on Wheels"[TW] OR Cooking[MH] OR Meals[MH] OR Food Services*[MH] ) AND ("experiences"[TW] OR "perceptions"[TW] OR "attitudes"[TW] OR "views"[TW] OR "difficulties"[TW] OR "needs"[TW] OR "habits"[TW] OR "behavior"[TW] OR "routines"[TW] OR Perception*[MH] OR Habits[MH] ) AND (2000/01/01:3000/12/31[dp])) NOT ("animals"[MH] NOT "humans"[MH]) NOT (Mice[MeSH] OR "Models, Animal"[MH])

Pubmed search strategy for eating (("older adults"[TW] OR "elderly"[TW] OR "seniors"[TW] OR "geriatrics"[TW] OR "aging"[TW] OR Aging*[MH] ) AND ("Community-dwelling "[TW] OR "Community-dwelling older adults "[TW] OR "Independent Living*"[TW] OR Independent Living*[MH] ) AND ("Mealtime"[TW] OR "Mealtime experiences "[TW] OR "Mealtime habits "[TW] OR "Eating habits "[TW] OR "Eating"[TW] OR "Eating motives"[TW] OR Eating*[MH] OR Meals[MH] OR Eating / psychology*[MH] ) AND ("experiences"[TW] OR "perceptions"[TW] OR "attitudes"[TW] OR "views"[TW] OR "difficulties"[TW] OR "needs"[TW] OR "habits"[TW] OR "behavior"[TW] OR "routines"[TW] OR Perception*[MH] OR Habits[MH] ) AND (2000/01/01:3000/12/31[dp])) NOT ("animals"[MH] NOT "humans"[MH]) NOT (Mice[MeSH] OR "Models, Animal"[MH])

1. Cairn search strategy

Cairn search strategy was as follow: Personnes âgées ET pratiques alimentaires OU Habitudes alimentaires ET Difficultés OU Besoins
